# Atlas of Legal Medicine

**Published:** 1899-12

**Authors:** 


**Atlas of Legal Medicine.** By Dr. E. VON HOFMANN. Edited by FREDERICK PETERSON, M.D., assisted by ALOYSIUS O. J. KELLY, M.D. London: The Rebman Publishing Co. (Ltd.). Philadelphia: W. B. Saunders. 1898.

Since the publication of this work in Vienna, Dr. E. von Hofmann, well known to medico-jurists, has joined the great majority, and the present translation and Englished edition of his work has appeared since his death.

The work is exactly what it purports to be; it consists of 56 plates in colours, and 193 illustrations in black and white. The letterpress descriptions given with the plates are good, sufficient, and to the point. The illustrations, with only one or two exceptions, are original, nor do they in any way repeat the usual stereotyped ones of the English books. They are excellently produced by process and in colour. Those in colour are worthy of all praise, and those that fail in any way owe their failure to the very great difficulty of the subject and not to the way in which the plate is produced. Plates 5—the respiratory organs and heart of a child at full term, 49—arsenical poisoning, and 54—*post-mortem* lividity, are particularly good. The plates illustrating the conditions of the hymen will prove most useful to the student who can have so little opportunity of practical knowledge on this point. The process plates of the ossification of the epiphyses of the lower extremities, (Figs. 79, 80), are most useful. We think that photographs of types of the insane and of idiots should have been given.

Instructive instances of the most important matters in medical jurisprudence are given. We trust our students will make good use of this excellent work.

